# Prevalence and correlates of suicidal behaviours among patients with depression and anxiety disorders in a multi-ethnic Asian setting

**DOI:** 10.3389/fpsyt.2026.1783251

**Published:** 2026-07-20

**Authors:** Anitha Jeyagurunathan, Edimansyah Abdin, S. Archana, Yeow Wee Brian Tan, Rajeswari Sambasivam, Shazana Shahwan, Jianlin Liu, Jayaraman Hariram, Nisha Chandwani, Steve Yin Lam Lee, Say How Ong, Poh Zhi Qian Brian, Yee Ming Mok, Mythily Subramaniam

**Affiliations:** 1 Research Division, Institute of Mental Health, Singapore, Singapore; 2 Department of Emergency and Crisis Care, Institute of Mental Health, Singapore, Singapore; 3 Department of Mood and Anxiety, Institute of Mental Health, Singapore, Singapore; 4 Department of Developmental Psychiatry, Institute of Mental Health, Singapore, Singapore; 5 Department of Psychology, Institute of Mental Health, Singapore, Singapore; 6 Assistant Chairman Medical Board (Clinical), Institute of Mental Health, Singapore, Singapore; 7 Saw Swee Hock School of Public Health National University of Singapore, Singapore, Singapore; 8 Lee Kong Chian School of Medicine, Nanyang Technological University, Singapore, Singapore

**Keywords:** anxiety, depression, outpatients, prevalence, SBQ-R, Singapore, suicidal behaviour

## Abstract

**Background:**

Suicidal behaviour refers to a spectrum of actions and thoughts focused on intentionally ending one’s own life, ranging from ideation and planning to suicide attempts. The study aims to (i) estimate the prevalence rate of suicidal behaviour among youths diagnosed with depression and anxiety disorders, using the Suicidal Behaviour Questionnaire (SBQ-R), (ii) evaluate the psychometric properties and factor structure of the SBQ-R as a measurement tool in the clinical population, (iii) identify the social, demographic and clinical factors which are associated with suicidal behaviour among youths with depression and anxiety in a multi-ethnic outpatient population in a tertiary hospital setting.

**Methods:**

379 participants who were seeking treatment for depression and anxiety disorders were recruited. The SBQ-R was used to identify the risk of suicidal behaviour. Data on socio-demographic variables, clinical variables, smoking status and alcohol status were also collected. Confirmatory factor analysis (CFA) was performed to establish the underlying factor structure of the SBQ-R. Multiple logistic regression were used for analyses.

**Results:**

The prevalence of suicidal behaviour in the past year was 84.4% in the sample. A CFA on the four items of the suicidal behaviour - indicated that the model fit the data well. The regression model showed that those of Indian ethnicity (vs Chinese ethnicity) were less likely to have suicidal behaviour, while current smokers (vs non-smokers), who had experienced childhood abuse (vs no childhood abuse), and those with PHQ-9 score of 10 and above were more likely to have suicidal behaviours.

**Discussion and conclusion:**

This study found that the SBQ-R has adequate psychometric properties for use among outpatients with depression and anxiety disorder in a tertiary hospital in Singapore. The high prevalence of suicidal behaviour in patients with depression and anxiety highlights the need for early intervention and targeted suicide prevention strategies in clinical settings.

## Introduction

Suicide is a major global public health concern with significant challenges across all societies and age groups. According to the World Health Organization (WHO), over 700,000 people die by suicide each year (1). Beyond being a personal tragedy, suicide represents a multifaceted social and mental health issue, ranking as the third leading cause of death among individuals aged 15–29 years (2, 3). Globally, suicide affects not only those who die by it but also families, communities, and healthcare systems, underscoring the urgent need for early identification, prevention, and intervention strategies. Suicidal behaviour encompasses a spectrum from thoughts to actions where an individual engages with intent to end their own life (4). It is a complex issue with several nuanced risk factors such as trauma, mental health conditions, substance abuse, and social isolation. On the other hand, suicidal resilience refers to the ability to withstand or bounce back from suicidal thoughts, feelings, or suicidal behaviour (5). It involves coping with adversity, stress, or trauma in a way that reduces the risk of suicidal behaviour (6). Understanding both risk and protective factors provides a comprehensive framework for suicide prevention, particularly within high-risk groups such as adolescents and individuals with mental illness.

Prior studies have found a range of demographic and clinical factors associated with an increased risk of suicide, including male gender, lower educational levels (7), unemployment, parental suicidal behaviours, psychiatric co-morbidities (8), and chronic physical disorders (9). Other studies have found that childhood trauma is linked to an increased suicide risk (10–12) and that patients with mood disorders were more likely to attempt suicide if they had experienced childhood trauma (13, 14). Among adolescents, Ati et al. (15) identified several interconnected risk factors, including depression, bullying, poor family relationships, low self-esteem, exposure to violence, and substance use. These risk factors often co-occur in a developmental stage characterized by identity exploration and heightened emotional reactivity, compounding the risk of suicidal behaviour. Similarly, numerous studies have demonstrated that having a psychiatric diagnosis confers a significantly higher risk of suicidal behaviour (16–18). A substantial proportion of individuals presenting to the emergency departments following suicide-related incidents are found to suffer from mood disorders (19, 20). Notably, a few studies identified that there are modifiable factors that protect against suicide among patients with depression and anxiety disorders (21, 22), reinforcing the close link between psychopathology and suicidal behaviour.

Youth is a period of significant change across life stages between childhood dependence to adult independence (23). Singapore defines youths as those aged 15–35 years (24). The nationwide Singapore Mental Health Study (SMHS) observed a higher prevalence of both mental disorders and suicidal behaviour among those aged 18–34 years (25, 26), and the recent Youth Mental Health Study in Singapore observed a high prevalence of psychological distress in youths (27). This is similar to what had been observed among youths and younger adults in other countries (e.g., The United States of America) (9). Locally, data from the Samaritans of Singapore (SOS) revealed that suicide was the leading cause of death among youths (10 to 29 years of age), with the number of suicide deaths amongst those aged 20 to 29 years being the highest compared to all other age groups (28). Similarly, a more recent study by Koh et al (29), examined individuals with suicidal ideation and found that 23.5% developed suicide plans and 15.9% made suicide attempts, with higher risk observed among those with psychiatric comorbidities, poorer perceived health, and social isolation. These local studies suggest that young people in Singapore are particularly susceptible to suicidal behaviour, emphasizing the need for early intervention and culturally contextualized prevention efforts.

While studies have identified risk factors for suicidality in the general population (12), such as gender, ethnicity, childhood maltreatment, there is a conspicuous absence of research in understanding suicidality among youth diagnosed with depression and anxiety disorders in Singapore. Furthermore, studies internationally have shown that suicidal experiences-including thoughts, plans and attempts are frequently concealed by patients and go unrecognized by families or healthcare providers and even when young people report them, their parents may invalidate them due to stigma, fear of hospitalization, or concerns about confidentiality (30–33). This hidden nature of suicidal experiences makes it imperative to identify and understand protective factors such as resilience, purpose in life, coping skills, perceived social support, and belongingness- as these may reduce the risk even when suicidal intentions are not overtly expressed. The current study was undertaken to bridge the identified research gaps and specifically aimed to (i) estimate the prevalence rate of suicidal behaviour among youths diagnosed with depression and anxiety disorders, using the Suicide Behaviour Questionnaire-Revised (SBQ-R), (ii) evaluate the psychometric properties and factor structure of the SBQ-R as a measurement tool in the clinical population, (iii) identify the socio-demographic and clinical factors which are associated with suicidal behaviour among youths with depression and anxiety in a multi-ethnic outpatient population in a tertiary hospital setting.

## Methods

The present study used data from a cross-sectional survey that was conducted at the Institute of Mental Health (IMH) between January 2024 and February 2025. IMH is the sole tertiary care psychiatric hospital in Singapore that serves a patient population with a wide range of mental illnesses. A total of 379 participants were recruited through convenience sampling. Patients seeking treatment at the IMH outpatient clinics were enrolled in the study. The study included patients who were seeking outpatient treatment and had a clinical diagnosis of depressive or anxiety disorder as determined by a psychiatrist using the Diagnostic and Statistical manual of Mental disorders, fourth edition (DSM-IV) criteria (34), were Singapore residents (including Singapore Citizens and Permanent Residents), aged 15 to 35 years, belonging to Chinese, Malay, Indian or other ethnic groups, capable of providing consent, and able to understand and read English. Patients who had intellectual disabilities or cognitive impairment or were not literate in English were excluded from the study. All participants provided written informed consent prior to their participation. Each participant took between 45 and 60 mins to complete the survey.

The study was initiated after receiving ethics approval from the relevant institutional ethics review board (National Healthcare Group Domain Specific Review Board (NHG DSRB ref: 2023/00589). The research was carried out in accordance with the Declaration of Helsinki and ethical principles of the Belmont Report.

### Measures

All participants completed a study questionnaire which included the following instruments:

Socio-demographic questionnaire: this included data on age, gender, ethnicity, marital status, education, employment status, and monthly personal income. Clinical variables such as current smoking and drinking status were collected from the participants.

To assess suicidal behaviour, the self-reported Suicidal behaviours questionnaire—revised (SBQ-R) (35) was used. The SBQ-R is composed of 4-items designed to identify the risk of suicidal behaviour in adolescents and adults. The sum of the items ranges from 3 to 18, with higher scores indicating greater suicidal risk. An SBQ-R score cut-off of 8 was used to create a non-suicidal behaviour (SBQ-R<8) and a suicidal behaviour (SBQ-R≥8) group (35). Internal consistency reliability of suicidal behaviour (Cronbach’s alpha =0.72) was acceptable in the current study.

The patient health questionnaire (PHQ–9) (36), is a 9-item self-administered instrument used to screen for the presence and severity of depression. Eight questions assess symptoms, and one question assesses functional impairment, all of which are scored on a scale of 0 to 3. A PHQ-9 score of 10 or more which was used as a cut off scores has also been shown to have a sensitivity of 88% and a specificity of 88% for identifying major depression (37). This scale has been widely used to measure the severity of depressive symptoms in psychiatric outpatient settings in Singapore (38). Internal consistency of PHQ-9 (Cronbach’s alpha =0.89) was acceptable in the current study.

The general anxiety disorder -7 (GAD-7) (39), is a 7-item self-administered instrument designed primarily as a screening and severity measure for generalized anxiety disorder, and has a score range of 0 to 21. A score of 10 or greater is recommended as a cut-off point for further evaluation when screening for anxiety disorders. Internal consistency of GAD-7 (Cronbach’s alpha =0.90) was acceptable in the current study. This scale has been widely used to measure the severity of anxiety symptoms in psychiatric outpatient settings in Singapore (38).

Childhood trauma questionnaire (CTQ) (40), measures severity of different types of adverse childhood events of which four were included in this study: Emotional abuse, physical abuse, emotional neglect, and physical neglect. The scale has been widely used in the clinical settings in Singapore (41–43). The original CTQ has 28 items but due to ethical concerns we had to drop the sexual abuse domain, and only 20 items were used in this study. For this study, emotional or physical abuse items specifically index experiences where caregivers inflicted harm- reflecting a violation of emotional or physical support-rather than mere absence of care (44).

### Statistical analysis

Statistical analyses were performed using Stata, version 18.0. Descriptive analyses were performed to establish the prevalence of suicidal behaviour (SBQ-R) as well as describe the sociodemographic profile of the study population. The construct validity of the 1-factor model of suicidal behaviour was examined using confirmatory factor analysis performed in R software using lavaan package. We used three criteria to indicate the goodness of fit of the factor structure of the suicidal behaviour: Comparative Fit Index (CFI), Root Mean Square Error Approximation (RMSEA) and Tucker-Lewis Index (TLI). The CFI and TLI both indicate a good fit if their values exceed 0.95 (45). Meanwhile, the RMSEA indicates adequate fit if it is < 0.08 (46), and good fit if the value is less than 0.05 (47). The reliability of the scale was evaluated using Cronbach’s alpha coefficient, in which the acceptable level was at least 0.7 (48). Bivariate analyses were conducted on the demographic and clinical variables to compare the two groups, i.e., suicidal behaviour and non-suicidal behaviour (SBQ-R cutoff score of 8). Subsequently a series of multivariate logistic regression analysis were performed to predict the suicidal behaviour score. Results were considered statistically significant if p-value was < 0.05.

## Results

Socio-demographic and clinical characteristics of the sample are presented in **Table 1**. The mean age of the participants (n=379) was 24.6, SD = 4.4 years, (range 15-35), with the majority being female (n=246, 64.9%), Chinese (n=267, 70.5%), single (n=347, 91.6%), with pre-tertiary education (n=200, 52.9%), and employed (n=201, 53.2%).

**Table 1:** Sociodemographic characteristics of the sample by SBQ-R (n=379).

Total sample			SBQ-R ≥ 8				
			No		Yes		
Mean age in years	Mean± SD		Mean± SD		Mean± SD		p value
	24.6± 4.4		25.4± 4.2		24.5± 4.4		
	N	%	N	%	N	%	
Gender							
Female	246	64.9	30	55.6	216	66.5	0.12
Male	133	35.1	24	44.4	109	33.5	
Ethnicity							
Chinese	267	70.5	37	68.5	230	70.8	0.621
Malay	64	16.9	8	14.8	56	17.2	
Indian	21	5.5	5	9.3	16	4.9	
Others	27	7.1	4	7.4	23	7.1	
Education							
Secondary completed (N/O level)	73	19.3	9	16.7	64	19.8	0.591
A level/polytechnic diploma	155	40.9	21	38.9	134	41.4	
Vocational certificate/ITE*	45	11.9	5	9.3	40	12.3	
University degree	106	28.0	19	35.2	86	26.5	
Marital status							
Married	26	6.9	4	7.4	22	6.8	0.97
Single	347	91.6	49	90.7	298	91.7	
Separated/widowed/divorced	6	1.6	1	1.9	5	1.5	
Employment							
Employed/Self-employed	201	53.2	32	59.3	169	52.2	0.586
Homemaker/Students	106	28.0	14	25.9	92	28.4	
Unemployed	71	18.8	8	14.8	63	19.4	
Personal income (SGD)*							
Below 2000	115	30.6	16	30.2	99	30.7	0.135
2000 to 3999	90	23.9	11	20.8	79	24.5	
4000 and above	54	14.4	13	24.5	41	12.7	
No income	117	31.1	13	24.5	104	32.2	
Smoking status							
Current smoker	61	16.1	2	3.7	59	18.2	**0.007**
Former smoker	55	14.5	5	9.3	50	15.4	
Non-smoker	263	69.4	47	87	216	66.5	
Alcohol status (past year)							
Never	220	58.2	37	68.5	183	56.5	0.115
Less than monthly	106	28.0	14	25.9	92	28.4	
Monthly/2-3times per month	52	13.8	3	5.6	49	15.1	
Patient health questionnaire (PHQ-9)							
No or mild depressive symptoms (PHQ-9<10)	104	27.4	30	55.6	74	22.8	**<0.001**
Moderate or severe depressive symptoms (PHQ-9 ≥ 10)	275	72.6	24	44.4	251	77.2	
Generalized anxiety disorder (GAD-7)							
No or mild anxiety symptoms (GAD-7 < 10)	157	41.4	35	64.8	122	37.5	**<0.001**
Moderate or Severe anxiety symptoms (GAD-7 ≥ 10)	222	58.9	19	35.2	203	62.5	
Suicidal behaviour questionnaire (SBQ-R)							
Suicidal behaviour (SBQ-R≥8)	325	85.8					
Non-suicidal behaviour (SBQ-R<8)	54	14.3					
Family history of attempted suicide in the past							
No	314	82.8	50	92.6	264	81.2	**0.04**
Yes	65	17.2	4	7.4	61	18.8	
Childhood trauma questionnaire (CTQ)_abuse							
No	82	21.6	24	44.4	58	17.8	**<0.001**
Yes	297	78.4	30	55.6	267	82.2	

ITE: Institute of Technical Education; SGD- Singapore Dollar. Bold numbers represent statistically significant values (p<0.05).

Of the sample, 16.1% (n=61) reported being current smokers and 14.5% (n=55) reported being former smokers. 28.0% (n=106) reported past year drinking of alcohol (less than monthly). In all, 72.6% (n=275) of the sample had a PHQ-9 score of 10 and above, while 58.6% (n=222) had a GAD-7 score of 10 and above.

A total of 97.3% of the participants reported having suicidal thoughts at least once in their lifetime and 84.4% had it in the past year. 29.8% told someone at least once in their lifetime, 29.6% stated never having told someone they had suicidal thoughts, while 40.6% told someone more than once about it. Notably, 32.2% said it was likely, rather likely or very likely that they would attempt suicide someday.

### Confirmatory factor analysis in the overall sample

**Figure 1:**
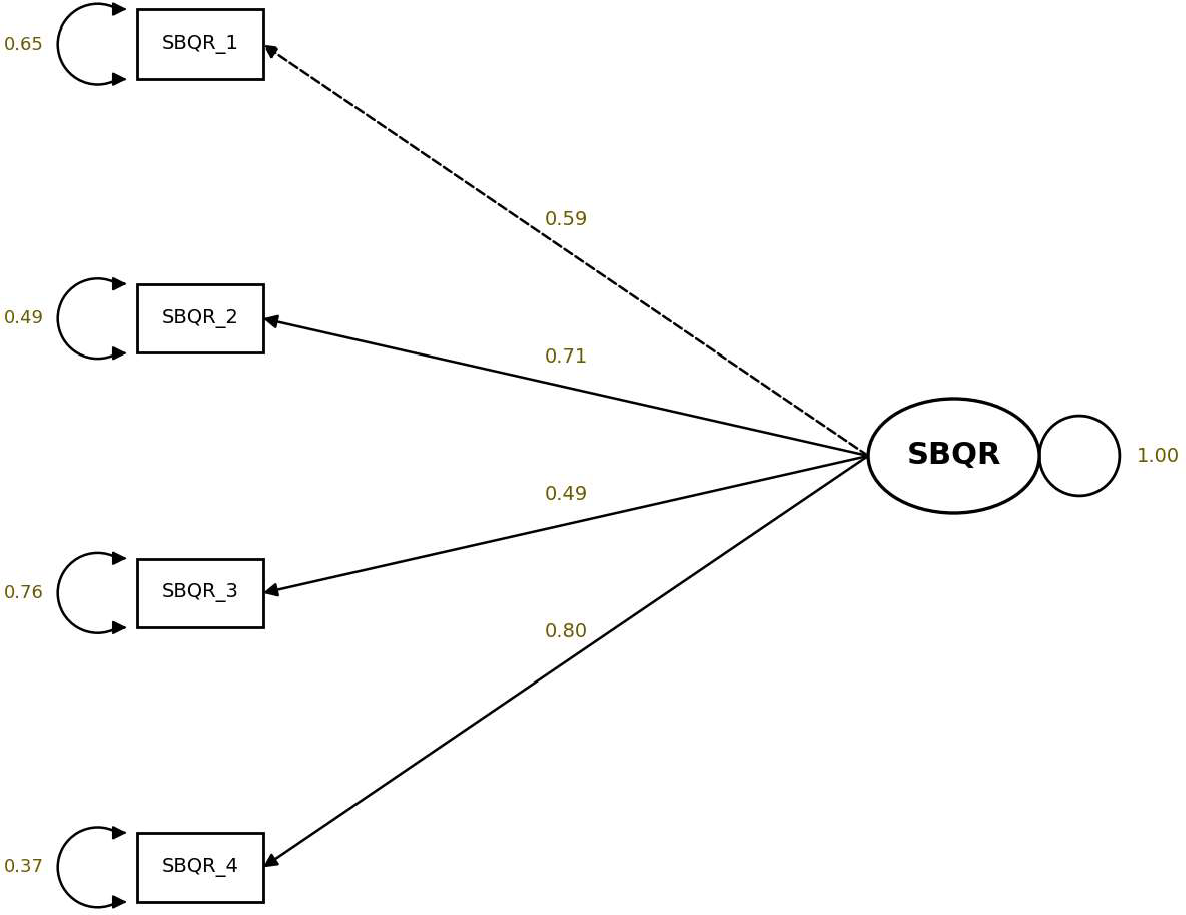
Confirmatory factory analysis of suicidal behaviour questionnaire (SBQ-R) path diagram.

The Cronbach’s alpha was 0.72. A confirmatory factor analysis on the four items of the suicidal behaviour indicated that the model fit the data well (**Figure 1**). All fit indices were good (CFI = 0.993; RMSEA = 0.061). Standardized factor loadings for items 1, 2, 3, and 4 were 0.59, 0.71, 0.49, and 0.80 respectively (**Table 2**).

**Table 2:** Standardized factor loadings and fit indices of the higher order one factor model.

Confirmatory factor analysis of suicidal behaviour questionnaire (SBQ-R)- 4 items		
Item	Standardized factor loading	
Item 1- Lifetime suicide ideation/attempt	0.59	
Item 2- Past 12-months suicidal ideation	0.71	
Item 3- Threat of suicide attempt	0.49	
Item 4- Suicidal behaviour in future	0.80	

Model fit statistics	Value	Cut off point
CFI (Comparative fit index)	0.993	>0.95
TLI (Tucker-Lewis index)	0.978	>0.95
RMSEA (Root mean square error of approximation)	0.061	<0.08
SRMR (Standardized root mean square residual)	0.037	<0.08

All standardized factor loadings were significant at p < 0.001.

### Socio-demographic and clinical correlates of suicidal behaviour

**Table 3** shows the results of the stepwise multivariate logistic regression models to assess the influence of different sets of predictors: Model 1 included only the sociodemographic factors. Model 2 added variables for childhood abuse, and family history of suicidal behaviours, while Model 3 included all previous variables plus severity of depression symptoms (PHQ-9) and severity of anxiety symptoms (GAD-7) (**Table 3**). In the fully adjusted regression model 3, those with childhood abuse (vs no childhood abuse), current smokers (vs non-smokers), and those with PHQ-9 score of 10 and above were more likely to have suicidal behaviour, while Indian ethnicity (vs Chinese ethnicity) remained less likely to have suicidal behaviour.

**Table 3:** Sociodemographic and clinical correlates of suicidal behaviour.

Variables	Model 1		Model 2		Model 3	
	OR (95% CI)	p-value	OR (95% CI)	p-value	OR (95% CI)	p-value
Mean age in years	0.94 (0.85-1.04)	0.239	0.95 (0.85-1.05)	0.303	0.96 (0.86-1.07)	0.464
Gender						
Female	1.56 (0.81-2.97)	0.181	1.45 (0.74-2.84)	0.276	1.16 (0.57-2.37)	0.676
Male	Reference		Reference		Reference	
Ethnicity						
Chinese	Reference		Reference		Reference	
Malay	1.01 (0.41-2.52)	0.982	1.11 (0.43-2.87)	0.831	0.92 (0.34-2.47)	0.871
Indian	0.30 (0.09-0.97)	**0.044**	0.31 (0.09-1.06)	0.063	0.20 (0.06-0.72)	**0.013**
Others	1.32 (0.35-4.95)	0.684	1.44 (0.37-5.52)	0.599	1.06 (0.26-4.27)	0.932
Education						
A level/polytechnic diploma	Reference		Reference		Reference	
Secondary completed (N/O level)	0.87 (0.33-2.28)	0.776	0.96 (0.36-2.57)	0.931	0.96 (0.35-2.63)	0.937
Vocational certificate/ITE*	0.82 (0.26-2.60)	0.734	0.88 (0.26-2.94)	0.84	0.94 (0.26-3.34)	0.92
University degree	1.13 (0.46-2.80)	0.793	1.19 (0.46-3.06)	0.721	1.45 (0.54-3.92)	0.459
Marital status						
Married	Reference		Reference		Reference	
Single	0.74 (0.20-2.68)	0.647	0.57 (0.15-2.21)	0.415	0.55 (0.14-2.13)	0.384
Separated/widowed/divorced	0.30 (0.02-4.22)	0.373	0.15 (0.01-2.25)	0.17	0.18 (0.01-2.94)	0.227
Employment						
Employed/self-employed	Reference		Reference		Reference	
Homemaker/students	1.12(0.40-3.10)	0.835	1.07 (0.37-3.11)	0.905	1.13 (0.39-3.28)	0.827
Unemployed	1.76(0.64-4.82)	0.271	1.84 (0.64-5.28)	0.26	1.60 (0.53-4.80)	0.406
Personal income (SGD)*						
No income	Reference		Reference		Reference	
Below 2000	0.73(0.27-1.97)	0.533	0.61 (0.22-1.74)	0.35	0.58 (0.20-1.68)	0.315
2000 to 3999	1.01 (0.31-2.30)	0.993	0.76 (0.22-2.61)	0.637	0.73 (0.20-2.59)	0.623
4000 and above	0.47 (0.14-1.65)	0.239	0.41 (0.11-1.56)	0.193	0.42 (0.11-1.68)	0.222
Smoking status						
Current smoker	7.21 (1.47-35.54)	**0.015**	7.50 (1.45-38.69)	**0.016**	8.07 (1.42-45.71)	**0.018**
Former smoker	2.29 (0.80-6.60)	0.122	1.97 (0.65-5.99)	0.231	2.17 (0.70-6.74)	0.179
Non-smoker	Reference		Reference		Reference	
Alcohol status (past year)						
Never	Reference		Reference		Reference	
Less than monthly	1.13 (0.54-2.39)	0.748	0.96 (0.44-2.09	0.917	0.87 (0.39-1.92)	0.73
Monthly/2-3times per month, weekly/2–3 times per week, and daily or almost daily	2.62 (0.68-10.07)	0.160	1.81 (0.45-7.31)	0.408	1.56 (0.36-6.71)	0.551
Childhood trauma questionnaire (CTQ)_abuse						
No			Reference		Reference	
Yes			3.49 (1.76-6.94)	**<0.0001**	2.62 (1.28-5.35)	**0.008**
Family history of attempt suicide in the past						
No			Reference		Reference	
Yes			2.16 (0.69-6.74)	0.185	1.83 (0.58-5.80)	0.305
Patient health questionnaire (PHQ-9)						
No or mild depressive symptoms (PHQ-9<10)					Reference	
Moderate or severe depressive symptoms (PHQ-9 ≥ 10)					2.54 (1.17-5.51)	**0.019**
Generalized anxiety disorder (GAD-7)						
No or mild anxiety symptoms (GAD-7 < 10)					Reference	
Moderate or severe anxiety symptoms (GAD-7 ≥ 10)					1.70 (0.77-3.76)	0.186

Model 1 – predictors: sociodemographic factors only.

Model 2 – predictors: sociodemographic factors, CTQ abuse and history of suicidal behaviour.

Model 3 – predictors: sociodemographic factors, CTQ abuse and history of suicidal behaviour, PHQ-9 and GAD-7. Bold numbers represent statistically significant value (p<0.05). Bold numbers represent statistically significant value (p<0.05). ITE: Institute of Technical Education; SGD- Singapore Dollar.

## Discussion

To the best of our knowledge, this was the first study to examine the prevalence and correlates of suicidal behaviour (using SBQ-R) in patients with depression and anxiety disorders in Singapore. The prevalence of suicidal behaviour in the past year was 84.4% in the sample. The SBQ-R demonstrated acceptable reliability and a unidimensional structure, supporting its use in this clinical population. Several significant correlates of suicidal behaviour were identified, including depression severity, smoking, and a history of childhood physical and emotional abuse. Ethnic differences were also observed, with Indian participants showing lower reported risk compared to Chinese participants. However, anxiety severity was not significantly associated with suicidal behaviour after adjusting for other factors. These findings were discussed in further detail in the subsequent sections.

### Psychometric properties and factor structure of the SBQ-R

In the present study, the SBQ-R demonstrated an acceptable internal consistency for use in individuals with depression and anxiety disorders. This value aligns closely with findings from similar clinical populations. For instance, Yook et al (50), reported an alpha of 0.74 among individuals with major depressive disorder, and Amini-Tehrani et al (51), found a value of 0.70 in patients with anxiety-related disorders. These comparable coefficients suggest consistent psychometric performance of the SBQ-R across varying psychiatric contexts. Notably, in earlier studies, e.g., Cotton et al (52), and Osman et al (35), reported slightly higher alpha values, ranging from 0.75 to 0.87, in samples involving clinical and non-clinical populations. This pattern supports prior observation that internal consistency may be slightly attenuated in clinical groups experiencing more severe or fluctuating symptomatology, due to cognitive or emotional factors affecting response reliability. Despite this variability, the SBQ-R maintains robust psychometric properties across populations, with all reported alpha values-including that of the current study, exceeding the widely accepted threshold of 0.70 (53), reinforcing its suitability for suicidal risk assessments in both research and clinical practice.

The current study’s results of the confirmatory factor analysis (CFA) conducted on the four items of the SBQ-R indicated that the one-factor model provided a good fit to the data, thereby supporting the unidimensional structure of the scale. This finding aligns with previous psychometric validations of the SBQ-R, which have consistently demonstrated that the instrument captures a single latent construct representing suicidal behaviour (35, 54).

### Socio-demographic and clinical correlates of SBQ-R scores

Those of Indian ethnicity were found to be at a lower risk of suicidal behaviour as compared to those of Chinese ethnicity. These findings were similar to that of a study conducted in Malaysia (55). In Singapore, Indians exhibit moderate suicide risk, lower than the Chinese population but higher than those of Malay ethnicity. National data indicate that Indians accounted for 38.4% of deaths due to accidents and violence, which includes suicides (56–58). In contrast, studies in India report higher suicidal risk, particularly among students, using the SBQ-R (59, 60).

An earlier study revealed that many Indians in Southeast Asia identify with Hinduism, Sikhism, or Islam, which often have strong religious prohibitions against suicide. These belief systems emphasize coping through spiritual support, karma, and collectivism, which may reduce suicidal behaviour (55). Studies found that religiosity and community bonding were stronger among Indian participants, which contributed to lower suicidal behaviours (61, 62). Cultural practices that emphasize collective coping and reliance on extended family networks may further strengthen these protective effects (58). Nonetheless, this apparent lower risk should be interpreted with caution, as the heightened stigma and taboo surrounding suicide in the Indian community may limit disclosure and result in underreporting (58, 62). On the other hand, Chinese participants, particularly younger adults may be more likely to acknowledge psychological distress, resulting in higher endorsement of suicidal thoughts and suicidal behaviours (59, 63). In another study from Singapore, Kok (63) noted a greater representation of Chinese female suicide attempters compared to other ethnic groups, suggesting cultural variation in help-seeking and disclosure patterns. The ethnic differences in suicidal behaviour among patients with depression and anxiety reflect a complex interplay of cultural, familial, religious, and psychological factors, which needs further research.

Current smokers were more likely to experience suicidal behaviour compared to non-smokers in the current study. Several epidemiological and clinical studies have consistently shown that smoking is associated with increased risk of suicidal ideation, attempts, and death, especially among individuals with mood and anxiety disorders (64, 65). The Singapore Mental Health Study- a nationwide epidemiological study, reported that smokers were more likely to report lifetime suicidal ideation and attempts, particularly those with comorbid depression or anxiety (66), this finding aligns closely with the current results. The consistent association between smoking and increased suicidal behaviour among individuals with depression and anxiety disorders highlights the need for integrated treatment (67). The possible explanation could be that nicotine alters the neurotransmitter systems- especially serotonin and dopamine- that are implicated in mood regulation. Dysregulation of these systems can worsen depressive symptoms and increase impulsivity, both of which are strong predictors of suicidal behaviour. Participants with depression and anxiety may use smoking as a coping mechanism for distress. It should be noted that the present study did not examine the potential dose-response relationship between smoking intensity and suicidal behaviour. While nicotine may provide short-term relief, it can worsen long-term mental health outcomes, creating a cycle of dependence and emotional dysregulation (64, 67). This association is evident across various populations and underscores the importance of addressing smoking habits in mental health interventions (68, 69). Incorporating smoking cessation programs into mental health care can potentially reduce both physical and psychological morbidity and serve as a critical component of suicide prevention strategies.

The present study found that individuals with a history of childhood physical and emotional abuse were significantly more likely to exhibit suicidal behaviour, even after controlling for sociodemographic and clinical variables. Emotional and physical abuse during early developmental stages have been shown to disrupt emotional regulation, cognitive appraisal, and interpersonal functioning- factors that are central to suicidal risk (8, 70). In the Singapore context, population-based data from the Singapore Mental Health Study revealed that individuals exposed to childhood maltreatment had significantly higher odds of developing mood and anxiety disorders, both of which are strong predictors of suicidal behaviour (12). Similarly, a local study among university students found high rates of emotional and physical abuse, which were significantly associated with depressive symptoms and suicidality (71). In Asian populations, the association between childhood abuse and suicidality has been well-documented (72, 73). These findings highlight the importance of screening for childhood trauma in clinical assessments and adopting trauma-informed interventions in mental health services aimed at reducing suicide risk. Systematic screening for childhood abuse using tools like CTQ may help clinicians identify patients at elevated suicide risk and tailor interventions accordingly.

Participants with moderate to severe depressive symptomatology had 2.5 times higher odds of suicidal behaviour than those with none or mild depression. The higher prevalence rate of suicidal behaviour among patients with depression can be attributed to several factors. Studies have shown that patients with depression often experience intense feelings of hopelessness, despair, and worthlessness, which can increase the risk of suicidal behaviour (17). Comorbid disorders can lead to a more complex symptom profiles, making treatment more challenging and increasing the risk of suicidal behaviour (49). Neurotransmitter imbalances such as serotonin and dopamine levels may further contribute to suicidal behaviour in patients with depression (74). Interestingly, while 58.6% of participants had a GAD-7 score of 10 and above (moderate to severe anxiety symptoms), after controlling for covariates in multivariate logistic regression, the direct association between GAD-7 scores and SBQ-R was no longer significant. We are unable to explain this finding and future studies in larger populations could help us understand if there are any other social or demographic factors in our population that have a more direct impact on suicidal behaviour as compared to severity of anxiety symptoms.

### Strength and limitations

The key strength of this study is its focus on a clinical population in Singapore, contributing locally relevant data on suicidal behaviour and supporting the validity of the SBQ-R in this context. However, several limitations should be acknowledged. This study was a cross-sectional study and limited to a single tertiary hospital; patients presenting to tertiary centres are more likely to have more severe or symptomatic conditions, so, it is not possible to generalize its findings to other settings. The cross-sectional study design did not allow the investigation of causal association of clinical factors with suicidal behaviour. The possibility of recall due to the use of self-administered questionnaires cannot be ruled out in the present study. The research focused on only four specific parameters of suicide-related thoughts and suicidal behaviours, as measured by the SBQ-R. This research did not examine the performance of the SBQ-R total scores and other suicide-related thought and suicidal behaviour parameters such as intent and self-harm. Future studies should adopt multi-centre, recruitment strategies across different levels of healthcare, include a wider age range, and apply probability-based sampling methods to enhance external validity and ensure more representative population estimates. The use of the SBQ-R cutoff score of 8, which was originally validated in Western clinical samples, may not fully account for cultural or contextual differences in Singapore or broader Asian populations. This could contribute to the high prevalence observed and reflects a limitation of the present study. Another limitation of this study is its reliance on DSM-IV criteria, which differ from DSM-5, potentially affecting diagnosis and comparability with recent studies. The study included only the use of ‘emotional abuse’ and ‘physical abuse’ items for analysis as childhood trauma. This modification would have led to the underestimation of overall trauma exposure and the weakening of associations between trauma and clinical outcomes. Lastly cultural or religious influences were not explored in sufficient depth to clarify their specific protective mechanisms. Future research should adopt longitudinal designs, include more diverse community samples, and incorporate qualitative approaches to better understand the cultural factors shaping suicidal behaviour across ethnic groups.

### Implications for practice and policy

These findings highlight the importance of early identification and comprehensive, integrated interventions for individuals with depression and anxiety disorders to reduce suicidal risk. Routine screening for suicidal behaviour, childhood trauma, and smoking should be incorporated into clinical practice. At the policy level, there is a need for broader, community-based mental health strategies, local validation of assessment tools, and targeted suicide prevention efforts that account for cultural and demographic differences within the Singapore population.

## Conclusion

In conclusion, this study found that the Suicidal Behaviour Questionnaire-Revised (SBQ-R) has adequate psychometric rigour for use among outpatients with depression and anxiety disorder in a tertiary hospital in Singapore. Its one-factor solution is consistent with other studies, and it had adequate internal consistency reliability. Those who were current smokers, had experienced childhood abuse, and with moderate to severe depressive symptoms were significantly associated with suicidal behaviour. The high prevalence of suicidal behaviour in patients with depression and anxiety highlights the need for early intervention and targeted suicide preventions strategies in clinical settings.

## Data Availability

The raw data supporting the conclusions of this article will be made available by the authors, without undue reservation.
